# Oral Cannabidiol Prevents Allodynia and Neurological Dysfunctions in a Mouse Model of Mild Traumatic Brain Injury

**DOI:** 10.3389/fphar.2019.00352

**Published:** 2019-04-16

**Authors:** Carmela Belardo, Monica Iannotta, Serena Boccella, Rosamaria Cristina Rubino, Flavia Ricciardi, Rosmara Infantino, Gorizio Pieretti, Luigi Stella, Salvatore Paino, Ida Marabese, Rosa Maisto, Livio Luongo, Sabatino Maione, Francesca Guida

**Affiliations:** ^1^Department of Experimental Medicine, University of Campania Luigi Vanvitelli, Naples, Italy; ^2^Enecta s.r.l., Bologna, Italy; ^3^Department of Plastic Surgery, University of Campania Luigi Vanvitelli, Naples, Italy; ^4^Drug Addiction Unit (SerT), Naples, Italy

**Keywords:** cannabidiol, traumatic brain injury, pain, behavior, microdialysis

## Abstract

Neurological dysfunctions are the most impactful and persistent consequences of traumatic brain injury (TBI). Indeed, previous reports suggest that an association between TBI and chronic pain syndromes, as well anxio-depressive behaviors, tends to be more common in patients with mild forms of TBI. At present, no effective treatment options are available for these symptoms. In the present study, we used a weight drop mild TBI mouse model to investigate the effect of a commercially available 10% Cannabidiol (CBD) oil on both the sensorial and neuropsychiatric dysfunctions associated with mild TBI through behavioral and biomolecular approaches. TBI mice developed chronic pain associated with anxious and aggressive behavior, followed by a late depressive-like behavior and impaired social interaction. Such behaviors were related with specific changes in neurotransmitters release at cortical levels. CBD oral treatment restored the behavioral alterations and partially normalized the cortical biochemical changes. In conclusion, our data show some of the brain modifications probably responsible for the behavioral phenotype associated with TBI and suggest the CBD as a pharmacological tool to improve neurological dysfunctions caused by the trauma.

## Introduction

The phytocannabinoid cannabidiol (CBD), the major non-psychoactive constituent of *Cannabis sativa*, exhibits a broad spectrum of potential therapeutic properties, including neuroprotective effects in Central Nervous System (CNS) disorders (Fernández-Ruiz et al., 2013; De Gregorio et al., 2018; Schonhofen et al., 2018). Through a multitarget mechanism, CBD shows potent anti-inflammatory and anti-oxidant properties which have been previously demonstrated in different models of neurodegenerative diseases and in acute episodes of brain damage (i.e., hypoxia-ischemia) (Hayakawa et al., 2007, 2010; Castillo et al., 2010). CBD has very low affinity for cannabinoid receptors type 1 (CB1) and type 2 (CB2), whereas different mechanisms, such as inhibition of anandamide uptake and enzymatic hydrolysis (Lastres-Becker et al., 2005), and decrease of adenosine reuptake (Carrier et al., 2006), are believed to be responsible for its neuroprotective effects.

Traumatic brain injury (TBI) is a complex injury with a number of symptoms accompanied by inflammatory process and cell death (Arciniegas, 2011; Liu et al., 2019). It is characterized by an initial neuroinflammation, mediated by a rapid glia cells activation, peripheral immune cells recruitment and secretion of inflammatory cytokines, followed by the late appearance of psychologically debilitating symptoms and cognitive impairments (Woodcock and Morganti-Kossmann, 2013). Despite recent advances in the knowledge of TBI pathophysiology, no adequate pharmacotherapies are currently available (Loane and Faden, 2010). It is assumed that the secondary neuropsychiatric changes that occur as a consequence of trauma are associated with plastic rearrangements at hippocampal and cortical circuitry (Schwarzbold et al., 2008). In these brain regions endocannabinoid (EC) molecules induce pro-homeostatic and neuroprotective effects, by affecting neuroplasticity in cognitive and affective processes (Vigano et al., 2009; Boccella et al., 2019). A growing body of evidence suggests that the pharmacological manipulation of EC attenuates neuroinflammation and improve the recovery of neurobehavioral function during the early weeks after TBI (Shohami et al., 2011; Mayeux et al., 2017; Schurman and Lichtman, 2017). To our knowledge, no study has evaluated the effects of CBD on the neurological dysfunctions associated with the TBI. In particular, we coupled behavioral tasks and biochemical evaluations to assess the CBD effects on long-term cognitive and emotional responses induced by trauma.

## Materials and Methods

### Animals

Male C57BL/6 mice (Charles River, Italy) weighing 18–20 g were housed three per cage under controlled illumination (12 h light/dark cycle; light on 6:00 A.M.) and standard environmental conditions (ambient temperature 20–22°C, humidity 55–60%) for at least 1 week before the commencement of experiments. Mice chow and tap water were available *ad libitum*. The experimental procedures were approved by the Animal Ethics Committee of University of Campania “L. Vanvitelli,” Naples. Animal care was in compliance with Italian (D.L. 116/92) and European Commission (O.J. of E.C. L358/1 18/12/86) regulations on the protection of laboratory animals. All efforts were made to reduce both animal numbers and suffering during the experiments.

## Mild TBI Induction

Experimental mTBI was performed using a weight-drop device developed in our laboratory. Mice were anesthetized with intraperitoneal injection of Tribromoethanol (250 mg/kg) and placed in a prone position on a spongy support. The head was not fixed. After a midline longitudinal incision, the skull was exposed to locate the area of impact and placed under a metal tube device where the opening was positioned directly over the animal’s head. The injury was induced by dropping a cylindrical metal weight (50 g), through a vertical metal guide tube from a height of 20 cm. The point of impact was between the anterior coronal suture (Bregma) and posterior coronal suture (Lambda). Immediately following injury, the skin was closed with surgical wound clips and mice were placed back in their cages to allow for recovery from the anesthesia and mTBI. Sham mice were submitted to the same procedure as described for mTBI, but without release of the weight. Sham and mTBI animals did not receive analgesic drugs after surgery. No animals have been excluded from the study.

### Drugs

Cannabidiol and vehicle were kindly provided by Enecta Group, Bologna (BO), Italy https://www.enecta.eu/?lang CBD was dissolved in hemp seed oil and natural tocopherols, used as vehicle. CBD (30 μl, oil 10%) was administered via gavage from day 1 to day 14 and from day 50 to day 60. Those time points represent the pathological windows in which we previously observed the main features of the mTBI, such as aggressiveness, recklessness and/or sensorial changes in the first phase, and the depressive-like behavior in a late phase (Guida et al., 2017a).

### Experimental Design

Time points of evaluations were based on our previous study (Guida et al., 2017a). A total number of 80 mice were divided in four experimental groups: Sham/vehicle, mTBI/vehicle, Sham/CBD and mTBI/CBD. Behavioral tasks were performed at different time points and scheduled in order to avoid carry-over effects from prior testing experience. The number of animals for each experiment is represented in Table 1.

**Table 1:** Numbers of animals used in each experiment.

Groups	Days					
	0	7	13–15	21	34	59–61
Sham/vehicle	*N* = 20	*N* = 12 (Pain) *N* = 10 (Rotarod)	*N* = 10 (Pain) *N* = 10 (Rotarod) *N* = 6 (Anxiety) *N* = 10 (Aggressive) *N* = 9 (Depression)	*N* = 5 (Pain) *N* = 5 (Rotarod)	*N* = 5 (Pain)	*N* = 5 (Rotarod) *N* = 3 (Sociability) *N* = 5 (Aggressive) *N* = 12 (Depression)
Sham/CBD	*N* = 20	*N* = 13 (Pain) *N* = 10 (Rotarod)	*N* = 10 (Pain) *N* = 10 (Rotarod) *N* = 6 (Anxiety)*N* = 10 (Aggressive)*N* = 9 (Depression)	*N* = 5 (Pain) *N* = 5 (Rotarod)	*N* = 5 (Pain)	*N* = 5 (Rotarod) *N* = 3 (Sociability) *N* = 5 (Aggressive) *N* = 12 (Depression)
mTBl/vehicle	*N* = 20	*N* = 13 (Pain) *N* = 10 (Rotarod)	*N* = 10 (Pain) *N* = 10 (Rotarod) *N* = 6 (Anxiety)*N* = 10 (Aggressive)*N* = 9 (Depression)	*N* = 5 (Pain) *N* = 6 (Rotarod)	*N = 5* (Pain)	*N* = 5 (Rotarod)*N* = 3 (Sociability)*N* = 5 (Aggressive)*N* = 12 (Depression)
mTBI/CBD	*N* = 20	*N* = 13 (Pain) *N* = 10 (Rotarod)	*N* = 11 (Pain) *N* = 10 (Rotarod) *N* = 6 (Anxiety) *N* = 10 (Aggressive)*N* = 9 (Depression)	*N* = 5 (Pain) *N* = 6 (Rotarod)	*N* = 5 (Pain)	*N* = 5 (Rotarod) *N* = 3 (Sociability) *N* = 5 (Aggressive.) *N* = 13 (Depression)


**FIGURE 1:**
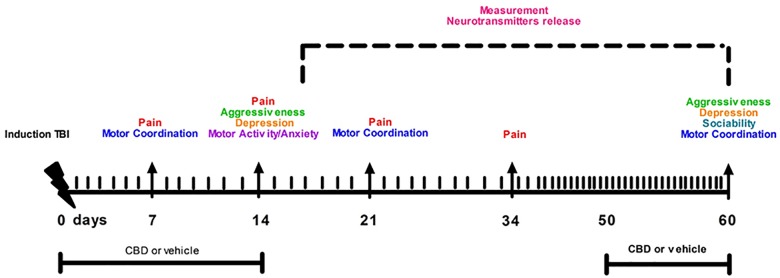
Timeline of mTBI induction, treatments and behavioral and biochemical characterization.

Following behavioral testing, mice were scarified for microdialysis experiments at 14 or 60 day after mTBI induction. The timeline of mTBI induction, treatments and behavioral and biochemical characterization is given in the Figure 1. Observers were blind to the treatments in each experiment.

### Tactile Allodynia

Tactile allodynia was evaluated at a series of calibrated nylon von Frey monofilaments (Semmes-Weinstein monofilaments, 2 Biological Instruments, Italy). Mice were allowed 7, 14 and 21 days after mTBI or sham surgery by to move freely in the compartment of the enclosure positioned on the metal mesh surface. Mice were adapted to the testing environment for 30 min before any measurement was taken. The monofilaments, starting from the 0.008 g monofilament, was applied perpendicularly to the plantar surface of each hind-paw in a series of ascending forces (0.008, 0.02, 0.04, 0.07, 0.16, 0.40, 0.60, 1.0, 1.4, 2.0, and 4.0 g). Each stimulus was applied for approximately 1 s with an interstimulus interval of 5 s. Withdrawal responses evoked by each monofilament was obtained from five consecutive trials. Data (gr) were expressed as a mean of right and left response hind paws. Voluntary movement, associated with the locomotion, was not counted as a withdrawal response. Tactile allodynia was defined as a significant decrease in the withdrawal threshold to the von Frey hair application. Each mouse served as its own control, the responses being measured both before and after surgical procedures.

### Rotarod Test

Possible motor coordination impairment was evaluated at 7, 21, and 60 days after mTBI or sham surgery by Rotarod test (Ugo Basile). Mice was measured for the time (s) of equilibrium before falling on a rotary cylinder by a magnet that, activated from the fall of the mouse on the plate, allows to record the time of permanence on the cylinder. After a period of adaptation of 30 s, the spin speed gradually increases from 5 to 40 rpm for a maximum time of 5 min. The animals were analyzed by two separate tests at 1-hour interval in the same day. The experiment was performed for every group of animals the day before the surgical procedure and the days before the behavioral tests in order to avoid stress. The time of permanence of the mouse on the cylinder was expressed as latency time (s).

### Open Field Test

Motor activity was also evaluated by open field test in sham and mTBI mice. Briefly, each mouse was individually monitored for 5 min in an open arena (l × w × h: 25 cm × 25 cm divided into 16 square grids). Parameters evaluated included: (1) number of transitions; and (2) number of rearings; and (3) time spent in the periphery or center (s).

### Resident-Intruder

At 14 and 60 days after mTBI or sham surgery, mice were tested for aggressive behavior using a resident intruder test. Mice were individually housed for 1 week in Plexiglas cages to establish a home territory and to increase the aggression of the resident experimental mice. To begin, food containers were removed and an intruder mouse of the same gender was placed in a resident home cage and resident–intruder interactions were analyzed for 10 min. The aggressive behavior of resident socially-isolated mice was characterized by an initial pattern of exploratory activity around the intruder, which was followed by rearing and tail rattle, accompanied in a few seconds by wrestling and/or a violent biting attack. The number of these attacks and latency to the first attack during the 10 min observation period was recorded.

### Three Chambers Sociability

Test at 60 days after mTBI or sham surgery, mice were tested for social interaction using a three-chambered social interaction apparatus. A plexi-glass three-chambered box was custom-built as follows: doorways in the two dividing walls had sliding covers to control access to the outer-side chambers. The test consisted of two consecutive stages of 5 and 10 min each. During the 5-minute first stage of habituation the mouse was allowed to freely explore the three chambers of the apparatus, detecting at this stage any innate side preference. After that the mouse was gently encouraged into the central chamber and confined there briefly by closing the side chamber doors. During the following 10-minute stage sessions, a custom made stainless-steel barred cup (6.5 cm × 15 cm) was placed upside down in one of the side chambers. A never before-met intruder, previously habituated, was placed into an upside-down identical cup in the other chamber. The time spent sniffing each upside-down cup, the time spent in each chamber and the number of entries into each chamber were recorded.

### Tail Suspension Test (TST)

The Depression like behavior was evaluated at 14 days and 60 days after mTBI or sham surgery, mice were individually suspended by the tail on a horizontal bar (55 cm from floor) using adhesive tape placed approximately 4 cm from the tip of the tail. The duration of immobility, recorded in seconds, was monitored during the last 4 min of the 6-minute test by a time recorder. Immobility time was defined as the absence of escape-oriented behavior. Mice were considered to be immobile when they did not show any body movement, hung passively and completely motionless.

### Microdialysis *in vivo*

**FIGURE 2:**
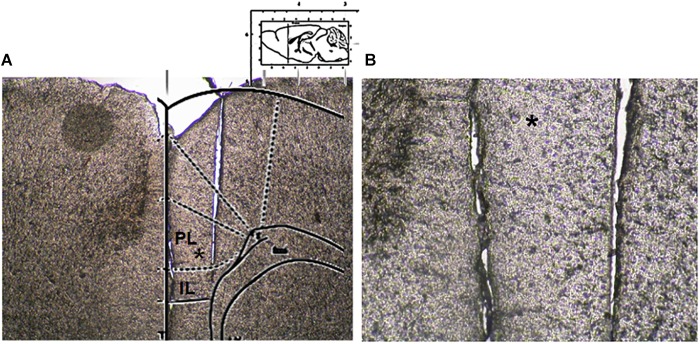
Microdialysis probe location. **(A)** Shows a panoramic picture of the pre-frontal cortex, the star indicates the prelimbic area. **(B)** Shows a high magnification of the microdialysis probe location for amino acid collection within the pre/infra-limbic cortex.

Microdialysis experiments were performed in awake and freely moving mice. In brief, mice were anesthetized with pentobarbital (50 mg/kg, i.p.) and stereotaxically implanted with concentric microdialysis probes into the mPFC using the coordinates: AP: 1.4–1.8 mm, L: 0.3–05 mm from bregma and V: 3.0 mm below the dura (Paxinos and Franklin, 2004). Dialysis probes, were constructed with 25G (0.3 mm inner diameter, 0.5 mm outer diameter) stainless steel tubing (A-M Systems). Inlet and outlet cannulae (0.04 mm inner diameter, 0.14 mm outer diameter) consisted of fused silica tubing (Scientific Glass Engineering). The probe had a tubular dialysis membrane (Enka AG, Wuppertal, Germany) 1.3 mm in length. Following a recovery period of 24 h, dialysis was commenced with aCSF (NaCl 147 mM, CaCl_2_ 2.2, KCl 4 mM; pH 7.2) perfused at a rate of 1 μL/min by a Harvard Apparatus infusion pump. The neurotransmitters release was evaluated after chronic treatment performed with CBD or Vehicle in m-TBI or sham animals. Data were expressed as the average of six repeated measurements (each 30 min) to give a more accurate value. No appreciable differences were observed between the different six dyslisate samples collected during the single experiment. At the end of experiments, mice were anesthetized and their brains perfused fixed via the left cardiac ventricle with heparinized paraformaldehyde saline (4%). Brains were dissected out and fixed in a 10% formaldehyde solution for 2 days and included in OCT compound. The brain was cut in 40-μm thick slices and observed under a light microscope to identify the probe locations (Figure 2). The concentrations of D-Aspartate, L-glutamate and GABA contained in the dialysate were analyzed using by HPLC coupled with fluorimetric detection method. The system comprised two Gilson pumps (model no. 303), a C-18 reverse-phase column, and a Gilson fluorimetric detector (model no. 121). Dialysates were pre-column derivatized with *o*-phthaldialdehyde-*N*-acetylcysteine (OPA-NAC) (10 μl dialysate + 5 μl OPA-NAC + 10 μl borate buffer 10%). The mobile phase consisted of two components: (A) 0.2 M Na_2_HPO_4_, 0.2 M citric acid and 20% methanol and (B) 90% acetonitrile. Gradient composition was determined using an Apple microcomputer installed with Gilson gradient management software. Mobile phase flow rate was maintained at 1.2 ml/min. Data were collected using a Dell Corporation PC system 310 interfaced to the detector via a Drew data-collection unit. Data were expressed as mean ± SEM of six samples for each mouse.

### Statistical Analysis

Data were represented as mean ± SEM. Behavioral data were analyzed by using one-way ANOVA, followed by Bonferroni’s multiple comparison. Newman–Keuls *post hoc* test was used as *post hoc* test in microdialysis analysis. *P* values < 0.05 were considered statistically significant. Statistical analysis was carried out using Prism/Graphpad (GraphPad Software, Inc.,) software. Numbers of animals used in each experiment is given in Table 1.

## Results

### CBD Effects on Allodynia in mTBI Mice

**FIGURE 3:**
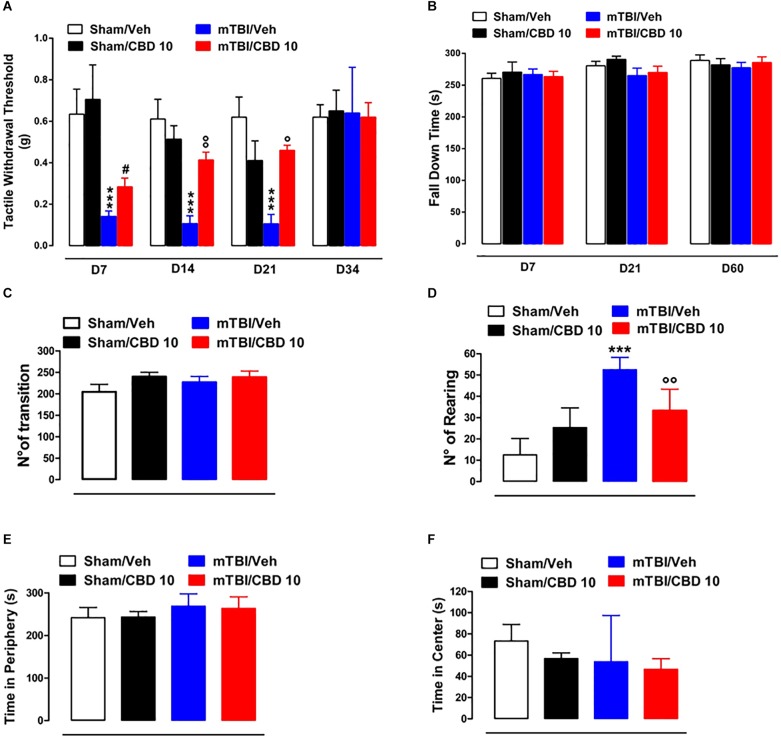
Effect of CBD on behavioral evaluations in sham and mTBI mice. **(A)** Shows Tactile withdrawal thresholds (TWT) measured through Von Frey monofilaments, **(B)** shows the latency to fall in the rotarod test, **(C–F)** show the number of transitions, number of rearing, the time spent in the periphery or in the center, in the open field test, respectively. Data are represented as mean ± SEM of 10–11 mice per group. **^∗^**, **^#^** and **°** indicate significant differences compared to sham/vehicle, sham/CBD 10 or TBI/vehicle, respectively. *P* < 0.05 was considered statistically significant. One-way ANOVA, followed by Bonferroni’s Multiple Comparison *post hoc* tests.

A significant decrease of TWT was observed in vehicle-treated mTBI mice at 7, 14, and 21 days after trauma induction [0.14 g ± 0.025, *F*(3,47) = 11.06, *P* < 0.0001; 0.11 g ± 0.04, *F*(3,37) = 12.40, *P* < 0.0001; 0.11 g ± 0.04, *F*(3,16) = 8.833, *P* = 0.0011, at 7, 14, and 21 days, respectively] compared to the sham group (0.63 g ± 0.12, 0.61 g ± 0.09, 0.62 g ± 0.1, at 7, 14, and 21 days, respectively) (Figure 3A). Moreover, a physiological re-establishment of normal pain response was observed 34 days after trauma induction [Sham/vehicle 0.62 g ± 0.06; Sham/CBD 0.65 g ± 0.1; mTBI/vehicle 0.64 g ± 0.22; mTBI/CBD 0.62 g ± 0.07 *F*(3,16) = 0.01345, *P* = 0.9978] (Figure 3A). No difference in pain threshold was observed between right and left paw (see Supplementary Table S1). Oral CBD treatment significantly reduced the tactile allodynia in mTBI mice at 14 and 21 days (0.28 g ± 0.04; 0.41 g ± 0.04; 0.46 ± 0.02, at 7, 14, and 21 days, respectively) as compared with vehicle (0.14 g ± 0.025; 0.11 g ± 0.04;0.11 g ± 0.04, at 7, 14, and 21 days, respectively) (Figure 3A). The CBD administration in sham mice did not change the pain response (0.71 g ± 0.17; 0.51g ± 0.07; 0.41g ± 0.09; 0.65 ± 0.1 at 7, 14 and 21, and 34 days, respectively) compared to sham/vehicle mice (0.63 g ± 0.12; 0.61 g ± 0.09; 0.62 g ± 0.1; 062 ± 0.06, at 7, 14, 21, and 34 days, respectively).

### CBD Effects on Motor Coordination and Anxiety in mTBI Mice

In the rotarod test, no difference in riding time was observed between any of the treatment groups (Figure 3B), indicating no impairments in motor coordination. In the open field test, used to assay general locomotor activity levels, but also anxiety, one-way ANOVA, followed by Bonferroni *post hoc* test, revealed no significant changes in the time spent in the center [sham/vehicle 73.33 s ± 15.63; sham/CBD 56.83 s ± 5.31; mTBI/ vehicle 53.80 s ± 19.51; mTBI/CBD 46.67 s ± 10.05, *F*(3,19) = 0.7663, *P* = 0.5269) or in the periphery [sham/vehicle 241.7 s ± 9.86; sham/CBD 243.2 s ± 5.31; mTBI/ vehicle 268.8 s ± 12.89; mTBI/CBD 263.3 s ± 11.12, *F*(3,19) = 1.896, *P* = 0.1646] or for the number of transitions [sham/vehicle 204.5 ± 17.50; sham/CBD 239.8 ± 10.41; mTBI/ vehicle 226.8 ± 13.62; mTBI/CBD 238.8 ± 14.06, *F*(3,20) = 1.354, *P* = 0.2854] after trauma or any treatment (Figure 3C,E,F). However, mTBI/vehicle [52.5 ± 2.38; *F*(3,20) = 24.00, *P* < 0.0001] mice showed an increase in the number of rearing as compared to sham/vehicle (12.50 ± 3.17) and this effect was significantly reduced by CBD treatment (33.33 ± 4.10). CBD did not change the number of rearing in sham animals (25.33 ± 3.77) (Figure 3D).

### CBD Effects on Aggressive Behavior in mTBI Mice

**FIGURE 4:**
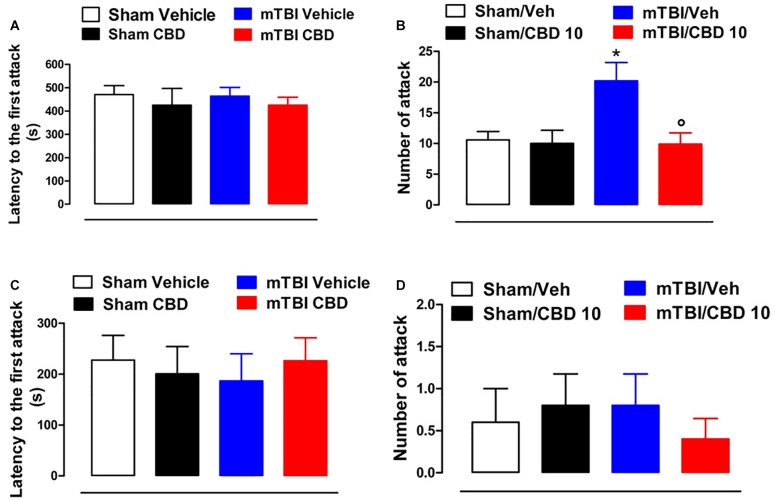
Effect of CBD on behavioral evaluations in sham and mTBI mice. **(A-D)** show the latency to the first attack and the number of attacks in the resident intruder test, respectively, at 14- and 60-days post mTBI. Data are represented as mean ± SEM of 10 mice per group. ^∗^ and ° indicate significant differences compared to sham/vehicle or TBI/vehicle, respectively. *P* < 0.05 was considered statistically significant. One-way ANOVA, followed by Tukey *post hoc test*.

No difference in the latency to the first attack in all groups of mice at 14 and 60 days after brain injury was observed [sham/vehicle 470.3 s ± 38.56; sham/CBD 424.6 s ± 71.80; mTBI/ vehicle 463.4 s ± 37.26; mTBI/CBD 424.5 s ± 34.10, *F*(3,36) = 0.2630, *P* = 0.8516; sham/vehicle 227.4 s ± 48.53; sham/CBD 200.4 s ± 53.56; mTBI/ vehicle 186.4 s ± 53.51; mTBI/CBD 226.0 s ± 45.33, *F*(3,16) = 0.1588, *P* = 0.9225] (Figure 4A,C). However, 14 days after the trauma, mTBI mice showed an increased number of attacks [20.20 ± 2.99, *F*(3,36) = 5.353, *P* = 0.0037], as compared to the controls (10.60 ± 1.36) (Figure 4B). CBD treatment significantly reduced this effect (9.9 ± 1.84) as compared with vehicle (20.20 ± 2.99). At 60 days after trauma, no significant change was observed in the number of attacks [sham/vehicle 0.6 ± 0.4; sham/CBD 0.8 ± 0.37; mTBI/ vehicle 0.8 ± 0.37; mTBI/CBD 0.4 ± 0.24, *F*(3,16) = 0.2933, *P* = 0.8296]. Sham mice treated with CBD did not show any change in the latency to the first attack or number of attacks compared to sham/vehicle mice (Figure 4C,D).

### CBD Effects on Depressive-Like Behavior in mTBI Mice

**FIGURE 5:**
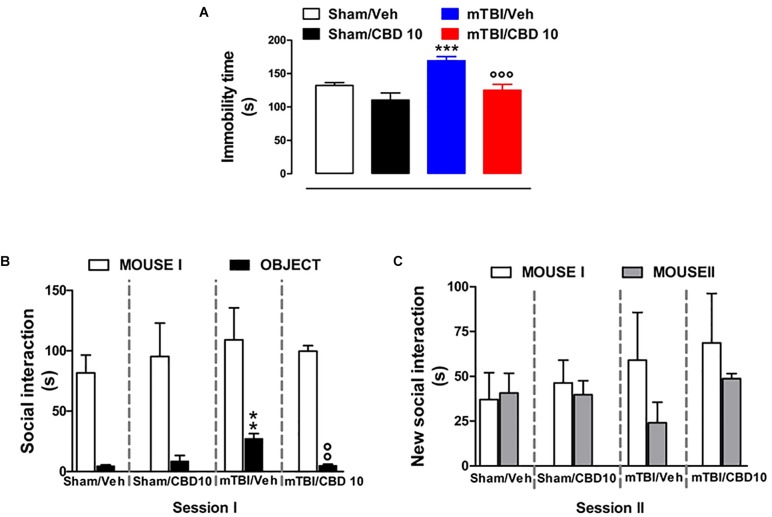
Effect of CBD on behavioral evaluations in sham and mTBI mice. **(A)** Shows the duration of immobility in the tail suspension test. **(B,C)** Show the duration of the time spent with an object or mouse in the three chambers sociability apparatus. Data are expressed in seconds and represented as mean ± SEM of 10–12 mice per group. ^∗^ and ° indicate significant differences compared to sham/vehicle or TBI/vehicle, respectively. *P* < 0.05 was considered statistically significant. One-way ANOVA, followed by Bonferroni’s Multiple Comparison *post hoc* test.

mTBI mice showed an increased immobility time, measured as the lack of escape-oriented activity (169.4 s ± 5.93) compared to the sham mice (132.4 s ± 4.15) 60 days post trauma (Figure 5A). CBD treatment significantly reduced the immobility in mTBI condition (125.1 s ± 8.95) compared to the vehicle (169.4 s ± 5.93) (Figure 5A). Sham mice treated with CBD did not show any change in the duration of immobility compared to vehicle-treated mice [110.4 s ± 10.53, *F*(3,45) = 13.64, *P* < 0.0001] (Figure 5A). At 14 days mTBI mice did not shown any change in the immobility time as compared with sham animals (Supplementary Figure S1).

### CBD Effects on Social Behavior in mTBI Mice

Analysis of the social preference revealed an impairment of social interaction which occurred 60 days post trauma. In the three chambers sociability test, no difference in the time spent in each chamber or in the number of transitions was observed in mTBI and Sham mice treated with vehicle or CBD (Supplementary Table S2). However, mTBI mice had reduced sociability level, spending a higher time in interacting with the object during the recorded time [27.33 s ± 4.1; *F*(3,8) = 11.40, *P* < 0.0029], compared to sham animals (4.67 s ± 0.88) (Figure 5B, session I). This effect was significantly improved in mTBI CBD-treated animals (5.0 s ± 1.0). Moreover, mTBI mice [interaction with mouse II: 24.0 s ± 11.55 *F*(3,8) = 1.308, *P* = 0.3373] did not show significantly altered preference for social novelty compared with control mice (interaction with mouse II: 40.67 s ± 11.05) (Figure 5C, session II). The CBD treatment did not induce any change in sociability, in the time spent in the two chambers or in the number of transitions between the chambers compared to vehicle in sham or mTBI animals (Sham/CBD and mTBI/CBD: interaction with mouse II: 39.67 s ± 7.86 and 48.67 ± 2.91) (Figure 5A,B).

### CBD Effects on Neurotransmitters Release mTBI Mice

**FIGURE 6:**
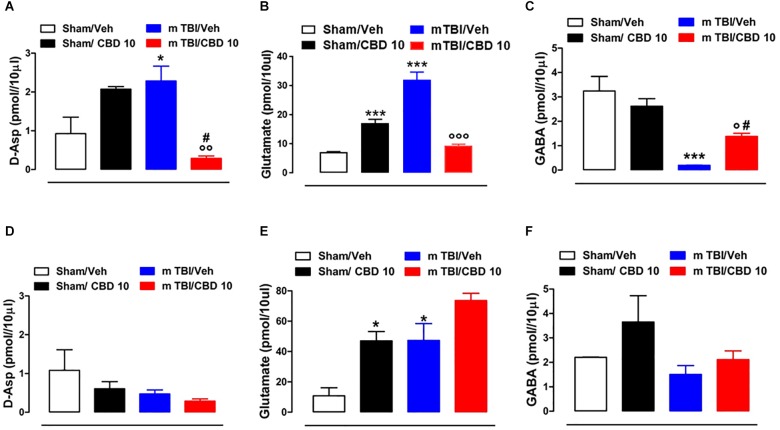
Effect of CBD on the release of glutamate **(B,E)**, GABA **(C,F)** and D-Aspartate **(A,D)** in sham or mTBI mice at 14 and 60 days after trauma. The values of extracellular amino acids in the mPFC were expressed as pmol in 10 μl of perfusate. Each point represents the mean ± SEM of 3–4 animals per group. **^∗^**, **^#^** and **°** indicate significant differences compared to sham/vehicle, sham/CBD 10 or TBI/vehicle, respectively. *P* < 0.05 was considered statistically significant. One- Way ANOVA, *post hoc* test Newman-Keuls Multiple Comparison.

*In vivo* microdialysis was used to assess the amino acids contents in the m-PFC of mTBI mice. HPLC analysis revealed a notable increase of extracellular glutamate (Glu) and D-Aspartate (D-Asp) levels in the mPFC of 14 days mTBI animals [Glu: 32.05 pmol/μl ± 1.33 *F*(3,12) = 123.1, *P* < 0.0001; D-Asp: 2.29 pmol/μl ± 0.38; *F*(3,8) = 7.922, *P* = 0.0088], as compared with controls (Glu: 7.12 pmol/μl ± 0.26; D-Asp: 0.93 pmol/μl ± 0.42). Remarkably, CBD treatment normalized both Glu and D-Asp levels (Glu: 9.43 pmol/μl ± 0.55; D-Asp: 0.28 pmol/μl ± 0.06) (Figure 6A,B). On the contrary, GABA levels were decreased by TBI, and CBD significantly reverted this effect (sham/vehicle 3.25 pmol/μl ± 0.6; sham/CBD 2.62 pmol/μl ± 0.31; mTBI/vehicle 0.191 pmol/μl ± 0.01; mTBI/CBD 1.38 pmol/μl ± 0.13, *F*(3,12) = 15.74 *P* = 0.0002) (Figure 6C). At 60 days post mTBI, while GABA and D-Asp dialysate were not changed [GABA: sham/vehicle 2.21 pmol/μl ± 0.01; sham/CBD 3.65 pmol/μl ± 1.08; mTBI/vehicle 1.51 pmol/μl ± 0.36; mTBI/CBD 2.11 pmol/μl ± 0.36, *F*(3,7) = 2.025 *P* = 0.1990; D-Asp sham/vehicle 1.08 pmol/μl ± 0.53; sham/CBD 0.61 pmol/μl ± 0.18; mTBI/vehicle 0.47 pmol/μl ± 0.10; mTBI/CBD 0.29 pmol/μl ± 0.06, *F*(3,7) = 2.176 *P* = 0.1789] (Figure 6D,F), Glu levels were still high, but CBD did not revert this effect [Glu: sham/vehicle 10.81 pmol/μl ± 5.33; mTBI/vehicle 47.29 pmol/μl ± 11.14; mTBI/CBD 73.62 pmol/μl ± 4.80, *F*(3,5) = 15.26 *P* = 0.0060] (Figure 6E). Finally, we found that CBD increased *per sè* Glu levels in the mPFC of sham mice at both 14- and 60-days post trauma (sham/CBD: 17.12 pmol/μl ± 1.40; 47.02 pmol/μl ± 6.09, 14 and 60 days, respectively), as compared with vehicle (sham/Vehicle: 7.12 pmol/μl ± 0.26; 10.81 pmol/μl ± 5.33, 14 and 60 days, respectively) (Figure 6B,E).

## Discussion

Cognitive and emotional dysfunctions are the most impactful and persistent consequences of TBI. Indeed, motor and sensory deficits and psychiatric disorders may endure for weeks, as a consequence of the traumatic damage to the underlying brain structures. We previously showed that mTBI induced late (up to 60 days) neurological dysfunctions in mice and identified electrophysiological changes at the cortical level possibly associated with symptomatology (Guida et al., 2017a). In the present study, we demonstrated that the repeated treatment with commercially available 10% CBD oil exerts beneficial effects on the behavioral dysfunctions associated with TBI. Moreover, at the dose tested, CBD does not change the normal attitude, in term of locomotion, nociception or emotional behavior, in not injured animals.

As previously shown (Guida et al., 2017a), 2 weeks-mTBI mice displayed abnormal pain response after innocuous stimuli to the paw (mechanical allodynia), probably due to the overall inflammatory condition (Feliciano et al., 2014). The daily treatment with CBD significantly reduced pain behavior, which, in fact, spontaneously disappeared in 30 days. Though brain trauma did not affect the motor coordination or the exploratory activity, we found an increased rearing activity in mTBI mice, which was counteracted by CBD treatment. The significance of rearing movements seems to be strongly related to the specific surrounding environment. Rearing may reflect attentive processes underlying the assembling of information in novel situations (Aspide et al., 1998), however, in some circumstances, it may simply represent an escape motivation (Lever et al., 2006). It is possible that the rearing activity in our model may reflect a kind of recklessness-like behavior, as previously reported in TBI mice (Guida et al., 2017a) and humans (DSM V). mTBI mice presented a typical phenotype, characterized by an aggressive behavior followed by a depressive-like behavior. Indeed, the aggressiveness of mTBI animals, revealed by an increased number of attacks on intruder mouse, was followed by a depressive-like behavior, manifested as enhanced immobility in the tail suspension test (14 and 60 days after trauma, respectively). The impaired social activity was also observed in the three-chamber sociability task, suggesting a general illness, often reported in patients with TBI. CBD significantly prevented all these effects. Interestingly, mTBI mice showed a reduced interest for social novelty, compared with controls. In fact, even if was not significant, we found that the time spent with the novel mouse (stranger) was reduced after trauma, indicating an impaired recognition memory. A positive trend to the increase was given by CBD treatment. TBI strongly affects the cortical neuronal plasticity. Indeed, we have previously shown that the altered behaviors following mTBI correlated with the biphasic firing activity of the pyramidal neurons in the mPFC, considered a key area regulating chronic pain (Giordano et al., 2011; Luongo et al., 2013) and negative affective states, such as anxiety and depression (Vialou et al., 2014; Apps and Strata, 2015; Guida et al., 2015). Remarkably, microdialysis/HPLC analysis revealed that mTBI (14 days) induced an increase of extracellular glutamate levels in the mPFC, which strengthens, the concept that plastic changes and novel neural remodeling may occur after trauma. Conversely, GABA levels were decreased, possibly as a counterbalance of the glutamate-mediated excitation. Remarkable, CBD normalized both GABA and glutamate levels. These latter data are in line with previous reports showing the protective effects of cannabinoids on the excitoxicity and inflammation correlated with glutamatergic system dysregulation in diverse neurodegenerative diseases (Guida et al., 2015, 2017b; Palazzo et al., 2015). In particular, the neuroprotective and antioxidant properties of CBD have been shown in high glutamate induced-toxicity in rat cortical neurons (Hampson et al., 1998). Our data also indicated that the extracellular levels of D-aspartic acid (D-Asp), endogenous NMDA receptor agonist, involved in pain and synaptic plasticity (Guida et al., 2015; D’Aniello et al., 2017) enhanced in mTBI mice. This effect was decreased in CBD-treated mTBI mice. Therefore, collectively these findings indicate that mTBI may be responsible for hyper-functional glutamate/D-aspartate signaling at the supraspinal level and, possibly, of the trauma-associated negative state (aggressive phenotype), at least at this time point. Indeed, while CBD reduced the depression and the impaired sociability, it was not able to change glutamate levels that were still high 60 days after trauma. This suggests the involvement of other brain areas, including the hippocampus, and/or other neurotransmissions (i.e., serotoninergic) in the altered neuropsychiatric behavioral profile of mTBI mice. Moreover, we demonstrated that CBD treatment also increased glutamate in sham mice, at both 14 and 60 days. Indeed, although it does not alter behavior in selected tasks, we cannot exclude that CBD may play a physiological role in other neuropsychological functions regulated by cortical processing, such as cognition, memory and reward.

## Conclusion

In conclusion, our data demonstrate that mTBI causes late sensorial affective/cognitive deficiencies linked to altered neurotransmitter release at cortical level. Moreover, we showed that chronic CBD treatment reduces behavioral dysfunctions by restoring at least in part cortical biochemical processes. Taken together, our results suggest that CBD could represent a novel approach for the management of neuropsychiatric disorders associated with TBI.

## Author Contributions

CB, LL, and RR conceived and designed the experiments. CB, MI, SB, FR, RI, and RM performed the experiments. GP, LS, SP, RR, LL, and IM analyzed the data and contributed to materials and analysis tools. CB, FG, and SM wrote the manuscript.

## Conflict of Interest Statement

RR is Enecta Group staff. CB is supported by a grant provided by Enecta Group. The experiments were not supported by Enecta. The remaining authors declare that the research was conducted in the absence of any commercial or financial relationships that could be construed as a potential conflict of interest.

## Supplementary Material

FIGURE S1 Effect of CBD on depressive-like behavior in sham and mTBI mice. The duration of immobility is measured in the tail suspension test at 14 days after mTBI induction.

TABLE S1 Effect of CBD on pain behavior in sham and mTBI mice. Left and right paw tactile withdrawal thresholds (TWT) are measured through Von Frey monofilaments at 7, 14, 21, and 34 days after mTBI induction.

TABLE S2 Effect of CBD on social behavior in sham and mTBI mice. The time spent in each chamber **(A)** or in the number of transitions **(B)** in the three chambers sociability apparatus.
