# Supplementary figures and images for: Oral Cannabidiol Prevents Allodynia and Neurological Dysfunctions in a Mouse Model of Mild Traumatic Brain Injury
Source: Front Pharmacol. 2019 Apr 16;10:352. doi: 10.3389/fphar.2019.00352 (PMC6476923; doi:10.3389/fphar.2019.00352)

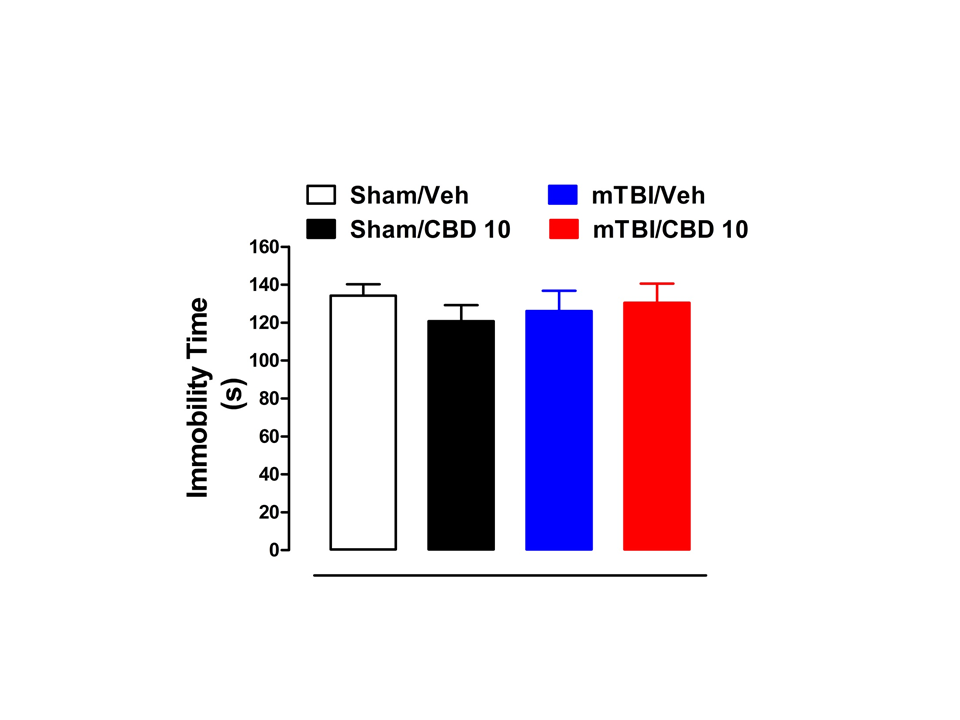

Supplement: FIGURE S1 — Effect of CBD on depressive-like behavior in sham and mTBI mice. The duration of immobility is measured in the tail suspension test at 14 days after mTBI induction. [file Image_1.TIF]

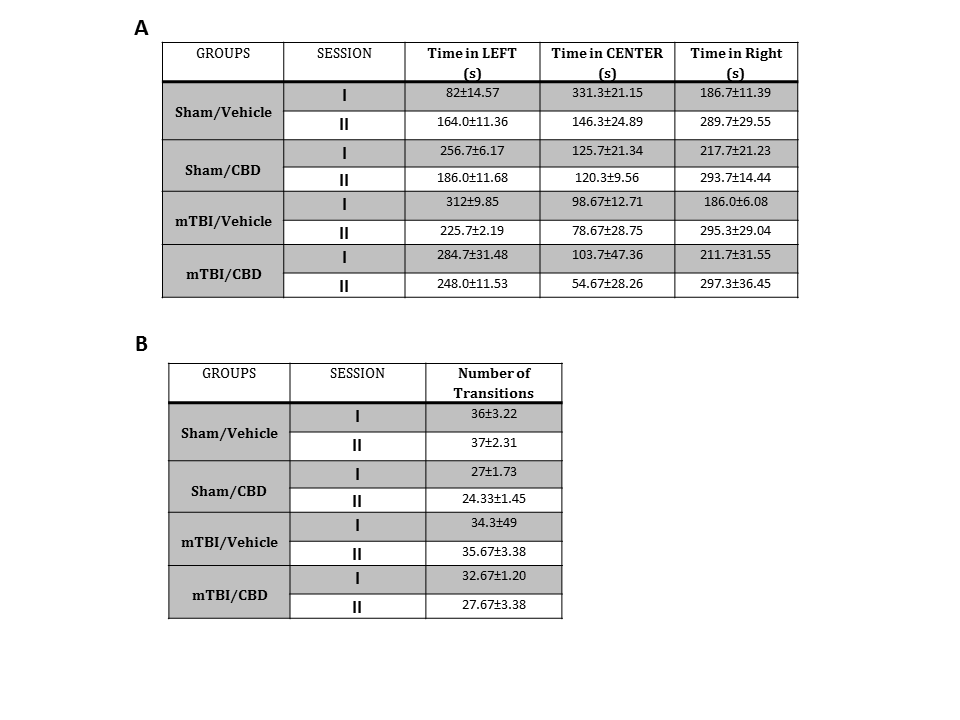

Supplement: TABLE S2 — Effect of CBD on social behavior in sham and mTBI mice. The time spent in each chamber (A) or in the number of transitions (B) in the three chambers sociability apparatus. [file Image_2.TIF]
