# Supplementary material for: Oral Cannabidiol Prevents Allodynia and Neurological Dysfunctions in a Mouse Model of Mild Traumatic Brain Injury
Source: Front Pharmacol. 2019 Apr 16;10:352. doi: 10.3389/fphar.2019.00352 (PMC6476923; doi:10.3389/fphar.2019.00352)
Supplement: TABLE S1 — Effect of CBD on pain behavior in sham and mTBI mice. Left and right paw tactile withdrawal thresholds (TWT) are measured through Von Frey monofilaments at 7, 14, 21, and 34 days after mTBI induction. [file Table_1.DOCX]

| Tactile Withdrawal Threshold (g) 7 days | | |
| --- | --- | --- |
| Groups | **Right (g)** | **Left (g)** |
| Sham/vehicle | 0.69 ± 0.18 | 0.57 ± 0.15 |
| Sham/CBD | 0.77 ± 0.17 | 0.65 ± 0.18 |
| mTBI/vehicle | 0.12 ± 0.03 | 0.15 ± 0.03 |
| mTBI/CBD | 0.30 ± 0.05 | 0.25 ± 0.04 |
| Tactile Withdrawal Threshold (g) 14 days | | |
| Groups | **Right (g)** | **Left (g)** |
| Sham/vehicle | 0.65 ± 0.18 | 0.57 ± 0.08 |
| Sham/CBD | 0.48 ± 0.09 | 0.55 ± 0.08 |
| mTBI/vehicle | 0.15 ± 0.06 | 0.06 ± 0.01 |
| mTBI/CBD | 0.44 ± 0.06 | 0.38 ± 0.05 |
| Tactile Withdrawal Threshold (g) 21 days | | |
| Groups | **Right (g)** | **Left (g)** |
| Sham/vehicle | 0.56 ± 0.11 | 0.68 ± 0.08 |
| Sham/CBD | 0.36 ± 0.09 | 0.45 ± 0.10 |
| mTBI/vehicle | 0.14 ± 0.06 | 0.07 ± 0.02 |
| mTBI/CBD | 0.44 ± 0.04 | 0.48 ± 0.04 |
| Tactile Withdrawal Threshold (g) 34 days | | |
| Groups | **Right (g)** | **Left (g)** |
| Sham/vehicle | 0.6 ± 0.10 | 0.64 ± 0.09 |
| Sham/CBD | 0.68 ± 0.13 | 0.62 ± 0.11 |
| mTBI/vehicle | 0.52 ± 0.20 | 0.76 ± 0.35 |
| mTBI/CBD | 0.56 ± 0.11 | 0.68 ± 0.13 |
